# Antimicrobial ethicists: Making ethics explicit in antimicrobial stewardship

**DOI:** 10.1017/ash.2021.181

**Published:** 2021-07-30

**Authors:** Christina F. Yen, James B. Cutrell

**Affiliations:** 1 Division of Infection Control and Hospital Epidemiology, Silverman Institute of Healthcare Quality & Safety, Beth Israel Deaconess Medical Center, Boston, Massachusetts; 2 Division of Infectious Diseases, Beth Israel Deaconess Medical Center, Boston, Massachusetts; 3 Division of Infectious Diseases and Geographic Medicine, Department of Medicine, University of Texas Southwestern Medical Center, Dallas, Texas

**Keywords:** Medical ethics, Antimicrobial stewardship, Antimicrobial resistance

## Abstract

Antimicrobial prescribing and the associated discipline of antimicrobial stewardship have inherent ethical and moral dimensions. We contend that the explicit, formal application of ethical principles and frameworks can strengthen and further justify the value of antimicrobial stewardship programs and their work. To illustrate the value of this process, we highlight 3 ethical scenarios that antimicrobial stewardship programs regularly encounter at the prescriber, institutional, and societal levels, and we analyze these scenarios using the Beauchamp and Childress biomedical ethics framework.

Antibiotic utilization from its inception has had moral and ethical dimensions. In Alexander Fleming’s oft-referenced 1945 Nobel Prize acceptance speech, he describes the hypothetical scenario of a patient’s death from penicillin-resistant streptococcal pneumonia, placing the moral responsibility for this death on the patient who inappropriately dosed penicillin during a prior infection.^1^ More than 75 years later, antibiotics remain a unique therapeutic class whose population-level efficacy is reduced with excessive and inappropriate use.^2^ The direct effect of this overuse—antimicrobial resistance (AMR)—has downstream epidemiologic, medical, financial, and societal consequences. The Centers for Disease Control and Prevention (CDC) 2019 Antibiotic Resistance Threat report estimates that 2.8 million antibiotic-resistant infections occur annually in the United States, resulting in 35,000 deaths and $4.6 billion in healthcare costs.^3^ The World Health Organization and the United Kingdom government have also published reports estimating that global annual AMR-associated deaths could be upward of 300 million by 2050 with an estimated additional cost of $1.2 trillion in healthcare expenditures.^4,5^ Although addressing AMR has mostly been reserved to the arenas of public health and health policy, the issue’s scope and complexity also has inherent ethical implications.^6–8^ Discussions regarding the ethics of AMR have focused primarily on the need for international surveillance, guidance, and policies to ensure equitable distribution of antibiotics where they are desperately needed while restricting them in countries or industries where indiscriminate use is prevalent. This call to action has included the need for increased support of antimicrobial stewardship programs. In fact, the term ‘antimicrobial stewardship’ was intentionally coined to emphasize its ethical nature, with ‘stewardship’ meaning the judicious usage and protection of a limited resource.^9,10^

Because antimicrobial stewardship carries this intrinsic ethical dimension, our contention is that a formal, explicit application of ethical principles and frameworks to antimicrobial stewardship would both strengthen the foundation of its work and highlight its societal value. To demonstrate how this can be applied, we have identified 3 ethical scenarios that stewards encounter regularly, spanning the individual, the institutional, and the societal levels. Although we recognize that these scenarios are not all encompassing, they are illustrative and serve as an invitation to more work and reflection in this space.

In our analysis of these scenarios, we apply the Beauchamp and Childress biomedical ethics framework to highlight the latent ethical considerations of these antimicrobial stewardship activities, drawing from both evidence-based medicine and ethical reasoning resources (Table 1).^11^ The Beauchamp and Childress framework is widely taught in graduate medical education and forms the basis for most modern ethical assessments in health care. It comprises 4 tenets: autonomy, justice, nonmaleficence, and beneficence. These can be briefly summarized as follows: (1) autonomy: the right for the patient—and in stewardship, we include the prescriber—to make medical decisions for themselves; (2) justice: balancing societal and individual good; (3) nonmaleficence: avoiding harm to patients; and (4) beneficence: acting not just to prevent harm but also to benefit the patient. These concepts resonate with our daily activities in health care, informing our recommendations and outcomes of interest. One limitation of this framework is that its principles may be interpreted by some as binary “yes or no” variables, rather than starting points for tackling more ethically ambiguous situations. As such, other frameworks can also be used to represent the spectrum and nuance of ethical reasoning, such as Kidder’s dynamics of ethical dilemmas: truth versus loyalty, individual versus community, short term versus long term, and justice versus mercy.^12^ Regardless of the framework used, an appreciation of when and how to apply ethical reasoning can strengthen the recommendations of antimicrobial stewardship programs and facilitate their desired outcomes.

**Table 1.** Childress and Beauchamp Biomedical Ethical Framework Applied to 3 Common Antimicrobial Stewardship Scenarios

Brief Definition	Autonomy	Justice	Nonmaleficence	Beneficence
	The right for a patient or prescriber to make healthcare decisions for themselves	Balancing benefits to patients and society with equity and impartiality	“Do no harm” or avoiding or minimizing harm whenever possible	Preventing harm by doing good; acting to promote the welfare of the patient
Scenario #1 Antimicrobial prescribing pressure (prescribers, patients)	-Is the intervention encroaching upon the prescriber or patient’s right to initiate, change, discontinue, or refuse antimicrobials? -Are the patient and prescriber making an informed decision about antimicrobial usage?	-Does the intervention favor a specific patient or patient population? -Does the intervention unfairly limit antimicrobial access to stakeholders who may need them?	-How does the intervention reduce patient or prescriber harm? -How does the intervention potentially expose a patient or prescriber to harm?	-Does the intervention increase the prescriber’s ability to help the patient? -Does the intervention help the patient make a better decision? -Does the intervention provide other benefits?
Scenario #2 Preauthorization/prospective audit and feedback	-To what extent is preauthorization limiting prescriber choice? -To what extent can or should AS programs manage “outlier” prescribers’ behavior?	-Does the intervention favor or discriminate against certain stakeholders? -How does the intervention impact the institution or society? -Do our metrics accurately measure appropriate use?	-How does the intervention of interest harm different stakeholders? -What harms do we risk if we defer the implementation of an intervention? -Are interventions reducing truth telling by incentivizing prescribers to lie or misrepresent their case?	-Do preauthorization and prospective audit and feedback benefit the prescriber? The patient? The AS program? -Does the intervention benefit the patient–prescriber or prescriber–AS program relationship?
Scenario #3 The individual versus the group or society	-To what extent can or should an intervention restrict a prescriber or patient who is acting against the interest of the group? -To what extent can an individual prescriber or patient limit the interests of the group?	-Does the intervention favor a particular stakeholder? -How do we measure and define acceptable risk or benefit to the individual and the group in the intervention? -What is the “opportunity cost” if resources are used for this intervention instead of others?	-What are the immediate and potential risks of this intervention to the patient, prescriber, or society at large? -Are certain stakeholders being placed at a higher risk than others? -Who is harmed to a greater extent if an intervention is not implemented?	-What immediate or future benefits could this intervention have for an individual patient? -What immediate or future benefits could this intervention have for a hospital or society at large?

Note. AS, antimicrobial stewardship.

## Scenario one: The prisoner’s (or prescriber’s) dilemma

A classic game theory scenario, the “prisoner’s dilemma” is a situation during which 2 accomplices are accused of a crime.^13^ Both are offered the chance to give up their partner in exchange for a reduced sentence, but by doing so, their accomplice will serve a longer one. To “win,” both people must agree in advance not to give each other up, thereby minimizing the consequences for both parties. This dilemma highlights how people, when given the opportunity, prefer communication and cooperation to reduce risk and maximize benefit. We can translate this theory into inpatient or outpatient medical practice with the “prescriber’s dilemma.” Prescribers believe patients are seeking antimicrobials and vice versa without first communicating their shared goals: finding a diagnosis and alleviating a patient’s suffering.^14^ The unspoken assumptions carried by both prescribers and patients of the other party’s desire for antimicrobials have a direct impact on prescribing and introduce additional ethical questions. Should the prescriber or patient’s autonomy supersede the communal need to reduce antimicrobial resistance? Is a narrow-spectrum antimicrobial, or none at all, an acceptable level of risk to the patient and prescriber? How can stewards serve as allies rather than the perceived “antibiotic police” to help both parties reach the “win” of judicious antibiotic prescribing?

One example of an antimicrobial ethicist’s potential solution to the prescriber’s dilemma is illustrated by a contemporary outpatient antimicrobial stewardship randomized clinical trial by Meeker et al.^15^ Of the 14 enrolled clinicians who practiced at 5 Los Angeles clinics, 7 clinicians displayed a public commitment poster, printed in English and Spanish and written at an eighth-grade reading level, signed by the clinic’s provider promising to prescribe antibiotics judiciously. This low-cost intervention resulted in an absolute reduction of inappropriate antimicrobial prescriptions for acute respiratory infections without a reduction in appropriate prescriptions. Furthermore, by implementing what the authors called a “nudge,” a term first coined by behavioral economists, they helped create an environment that facilitated ethical decision making.^16^ The intervention successfully enables both parties to ‘win’ the dilemma by ensuring shared decision making that prioritizes the bioethical values of beneficence and nonmaleficence. It also limits antimicrobial stewardship encroachment on prescriber and patient autonomy and confers societal and individual benefits (justice). By tapping into the preference for cooperation and communication, other antimicrobial stewardship interventions that leverage sociobehavioral insights into prescribing practices can promote more ethical stewardship ‘wins’ through shared decision making.^17^

## Scenario two: Preauthorization and prospective audit and feedback

In the 2016 Infectious Diseases Society of America (IDSA)/Society for Healthcare Epidemiology of America (SHEA) antimicrobial stewardship implementation guidelines, the ethical dimensions of the 2 core antimicrobial stewardship interventions, preauthorization and prospective audit and feedback, are described but not explicitly stated.^18^ Two potential ethical pitfalls of these interventions are identified in the guidelines: limitations to autonomy and the avoidance of truth telling. Even though truth telling is not featured in the Childress and Beauchamp framework, it is featured in clinical ethics and could be considered a component of all 4 tenets.^19^ As it pertains to antimicrobial stewardship, the goal is to create an environment that supports clinician truth telling about their patients and discourages “bypassing” indications or withholding information that may limit the effectiveness of antimicrobial stewardship interventions. To ensure that antimicrobial stewardship programs are not acting capriciously or in an unethical manner by excessively limiting prescriber autonomy or compromising truth telling, preauthorization or audit-and-feedback activities ideally should be guided by consensus guidelines or metrics of appropriate antimicrobial use.

However, even defining and measuring what constitutes “appropriate use” for the purposes of preauthorization and prospective audit and feedback challenges our conceptions of what harm-avoidant, beneficent prescribing means. Commonly used metrics (e.g., days of therapy or defined daily doses) describe consumption in excess or deficit which may suggest possible harm, but they cannot directly gauge proper usage. Additional layers of information such as diagnosis indication, local antibiograms, and unit or peer comparisons can also provide additional clues about the appropriateness of prescribing. Finally, the standardized antimicrobial administration ratio (SAAR), which parallels the standardized infection ratio (SIR), provides external benchmarking to highlight potential areas of inappropriate usage but, again, cannot speak conclusively to appropriateness.^20^ Therefore, continued work by the antimicrobial stewardship community is needed to establish better metrics for appropriate usage. This process will also encourage truth telling by providers and will highlight the value of antimicrobial stewardship programs to various stakeholders.

Two new areas of infectious diseases and antimicrobial stewardship research offer promise in this regard. First, novel clinical trial designs using more holistic definitions and measurement of appropriate usage can provide the information we need to make more robust scientific and ethical decisions. For example, the desirability of outcome ranking (DOOR) methodology challenges the binary outcomes of “cured or not cured, survival or mortality” by ranking the spectrum of potential outcomes based on an integrated assessment of benefits and harms. This approach places value on the treatment itself, which aligns with deontological (virtue) ethics, not just the outcome, which aligns with teleological (consequentialist) ethics.^20,21^ DOOR thereby strives to provide healthcare professionals the resources to make pragmatic, ethically nuanced decisions that balance the benefits and harms of therapeutic options. Second, the field of qualitative or mixed method antimicrobial stewardship studies can help identify ethical components and tensions latent to stewardship interventions. For example, Charani et al.^22^ conducted an ethnographic survey of surgical and medical team prescribing practices elucidating key differences in perceived autonomy, decision making, and responsibility, all of which shed light on behavioral and ethical intervention opportunities.^23^ Ultimately, to maximize the effectiveness of preauthorization and audit and feedback, a critical appraisal of antimicrobial stewardship metrics, clinical trial designs, and the antimicrobial prescribing “culture” of an organization are needed to deploy antimicrobial stewardship interventions in the most effective and ethical manner.

## Scenario three: The runaway (antimicrobial) trolley

Whereas the “prisoner’s or prescriber’s dilemma” only involves two stakeholders, the classic ethical dilemma of “the runaway trolley” asks us to consider how to justify the moral and ethical decision to save 1 person versus 5 people. In its most rudimentary form, a runaway trolley is careening down the tracks toward 5 bystanders; if left to run, it will hit and kill all 5 individuals. However, the trolley can be diverted with the flip of a switch to another track, where 1 bystander is located. The act of flipping the lever does mean that 1 individual will be hit by the trolley and killed.^24^

“The runaway trolley” dilemma does not regularly play out in such extreme terms in routine medical practice. However, it highlights how ethical theories such as utilitarianism (saving 5 individuals means quantitatively saving 4 more lives than you would have if the trolley were not diverted) can play out in antimicrobial stewardship decisions at an institutional or societal level. Choosing and implementing interventions that will benefit or are enacted at the collective level versus the individual level is analogous to the choice between the 5 bystanders versus the 1. These collective approaches are embedded in national campaigns to eliminate low value care (e.g., the Choosing Wisely campaign).^25^ Another emerging example on a local level is the antimicrobial stewardship concept of expected practice, which seeks to delineate shared institutional expectations for clinical care. Expected practices are meant to be more robust and institutionally tailored than professional society guidelines by securing the buy-in of hospital leadership and citing the latest local data. Additionally, the act of achieving practice consensus and codifying collective expectations also signals institutional buy-in to champion beneficence and nonmaleficence through ethical clinical decision making. In the case of Yadav and colleagues’ expected practice regarding shorter antimicrobial duration for common bacterial infections, prescribers who had previously expressed concerns regarding limits on autonomy with other antimicrobial stewardship interventions did not feel so with the implementation of expected practices, and shorter, evidence-based therapeutic courses were observed.^26^

Another illustration of “The Runaway (Antimicrobial) Trolley” dilemma was seen in the recent IDSA Sepsis Task Force’s decision to decline support of the latest Surviving Sepsis campaign guidelines.^27^ Although clinical implementation concerns were raised, the IDSA Sepsis Task Force also raised ethical concerns, although they were not explicitly identified.^28^ One example was the absence of stewardship considerations in antimicrobial treatment recommendations, such as empiric “combination therapy” for the presumed presence of multidrug-resistant organisms if a patient was in sepsis or septic shock. The IDSA Sepsis Task Force cited concerns for individual patient harm (failing to ensure nonmaleficence) that outweighed the potential, theoretical benefits proposed by the Surviving Sepsis campaign. At an ecologic level, these concerns were related to the acceleration of the development of antimicrobial resistance by placing undue emphasis on individual prescriber judgment without prescribing oversight or feedback (i.e., compromising justice for the sake of autonomy).

However, we must remember that the ethical nature of an antimicrobial stewardship intervention is dynamic. For the discipline’s continued maturation, it is thereby necessary to continue reapplying ethical frameworks to emerging scientific data. Just over a decade ago, the concept of shorter antimicrobial therapy durations was considered dangerous and even unethical given a lack of well-designed studies and concerns for causing more patient harm than benefit. In his landmark 2008 Maxwell Finland lecture, Dr. Louis Rice challenged the infectious diseases community to re-examine this longstanding dogma related to antibiotic duration, highlighting the harms of prolonged courses such as AMR and *Clostridioides difficile* infection.^29^ By weighing the beneficence and nonmaleficence of long versus shorter courses of therapy, Dr Rice called for evidence-based and ethical clinical decision making rather than fear or ‘eminence-based’ medicine. Since then, multiple clinical trials spanning a broad range of common infectious diseases have confirmed that, with few exceptions, shorter durations of therapy do not incur greater patient harms while they achieve the same favorable outcomes seen with longer courses. These findings have been so compelling that they now feature in practice guidelines not only for infectious diseases specialists but also internists.^30^ This significant paradigm shift, driven by emerging scientific data, illustrates the ongoing need for ethical re-examination of antimicrobial stewardship best practices in the future.

In conclusion, antimicrobial stewards make daily decisions that are inherently ethical. However, the question remains: Should the ethical nature of this work be explicitly articulated or not? We strongly believe that there is real value in the recognition and articulation of the ethical dimensions of antimicrobial stewardship. First, an ethically informed approach to antimicrobial stewardship will align programs’ work with both CDC Core Elements and regulatory requirements that center on delivery of safe and effective patient care.^31^ Second, using a robust ethical framework to inform program priorities and implementation will enhance engagement with key partners in infection prevention and control, environmental health safety, employee and occupational health, and others in hospital leadership. Furthermore, such a framework makes the case for stronger institutional support and resources dedicated to antimicrobial stewardship.^32^ Third, an emphasis on the ethics of antimicrobial stewardship will naturally complement the application of behavioral frameworks in antimicrobial stewardship to produce sustainable “culture change” within an institution.^33^ Fourth, these ethical imperatives can drive scientific research and innovation by which the evidence base in support of antimicrobial stewardship “best practices” can be advanced. However, none of these benefits will accrue passively. They will require both practical training in the application of biomedical ethics for antimicrobial stewards and active engagement on these topics within the antimicrobial stewardship community at large. Without doing so, we will lack the shared ethical resources and moral imperative to stem the tide of the looming global crisis of antimicrobial resistance—a slow-moving pandemic, marching relentlessly forward.
